# Psoralen induced cell cycle arrest by modulating Wnt/β-catenin pathway in breast cancer cells

**DOI:** 10.1038/s41598-018-32438-7

**Published:** 2018-09-18

**Authors:** Xiaohong Wang, Chengfeng Xu, Yitong Hua, Kai Cheng, Yingzhe Zhang, Jian Liu, Yong Han, Song Liu, Guoqiang Zhang, Shujian Xu, Zhenlin Yang

**Affiliations:** Department of Thyroid and Breast Surgery, Binzhou Medical University Hospital, Binzhou, Shandong 256603 P. R. China

## Abstract

Psoralen could inhibit the proliferation of human breast cancer cells, however, the molecular mechanism was unclear. We evaluated the anti-proliferative effects of psoralen by MTT, plate colony formation assay and cell cycle analysis in MCF-7 and MDA-MB-231 cells. The effects of psoralen on activation of Wnt/β-catenin and the related target genes were examined by quantitative real-time PCR, western blotting and cell immunofluorescence. The tumor growth was conducted in BALB/c nude mice and the pathological changes of heart, liver and kidney were also observed. Our results demonstrate that psoralen significantly inhibited cell proliferation by inducing G0/G1 phase arrest in MCF-7 cells and G2/M phase arrest in MDA-MB-231 cells. The expression of Fra-1 was reduced and Axin2 was promoted both in MCF-7 and MDA-MB-231 cells after psoralen treatment. The cytoplasmic accumulation and nuclear translocation of β-catenin were significantly reduced by psoralen. Psoralen increased the levels of phospho-(Y142) β-catenin, while decreased the expression of total β-catenin and its downstream target Fra-1 *in vitro* and vivo. Moreover, psoralen didn’t cause any significant toxicity at the effective concentration. Overall, our results might provide theoretical basis for clinical application of psoralen in breast cancer.

## Introduction

Breast cancer is the most common form of cancer in Chinese women^1^. The main characteristic of breast cancer is uncontrollable proliferation^2^. Therefore, blocking the cell cycle is regarded as an effective strategy for eliminating cancer cells. Since 1982 and the initial discovery of Int1 (Wnt1a), an oncogene in murine breast cancers^3^, Wnt signaling has been strongly associated with cancer cell proliferation through regulation of the cell cycle.

The canonical Wnt/β-catenin pathway plays a pivotal role in regulating tumorigenesis by arresting the cell cycle at different phases. When β-catenin is stabilized, it accumulates in the nucleus and constitutively activates its cell cycle-related target genes, such as c-Myc, cyclin D1, p16, PPARγ and Fra-1. Functionally, Fra-1 can promote tumor cell proliferation, inhibit apoptosis^4^, and increase cell invasion^5^ and vascular invasion^6^. Several recent observations have shown that Fra-1 not only has an essential role in breast tumorigenesis^7^ but also drives the expression of a highly prognostic gene set^8–11^. The QIAGEN transcription factor binding sites in the Fra-1 gene promoter include TBP, STAT1, p53, p300, ATF-2 and C/EBPα, which are all important for cell proliferation and cell cycle progression. In our previous studies, Fra-1 was significantly downregulated after psoralen treatment in human breast cancer MCF-7 and MCF-7/ADR cells. The anti-tumor effect of psoralen has been studied since 1959^12^; however, the anti-tumor mechanism is still unclear.

Based on our previous study, we analyzed the effect and mechanism of psoralen on cell proliferation and cell cycle progression mediated by the Wnt/β-catenin signaling pathway in MCF-7 and MDA-MB-231 cells. We also assessed the changes in other organs and provided useful information for balancing the safe and rational use of psoralen *in vivo*.

## Results

### Psoralen inhibit the proliferation, colony formation and cell cycle of breast cancer cell

**Figure 1:**
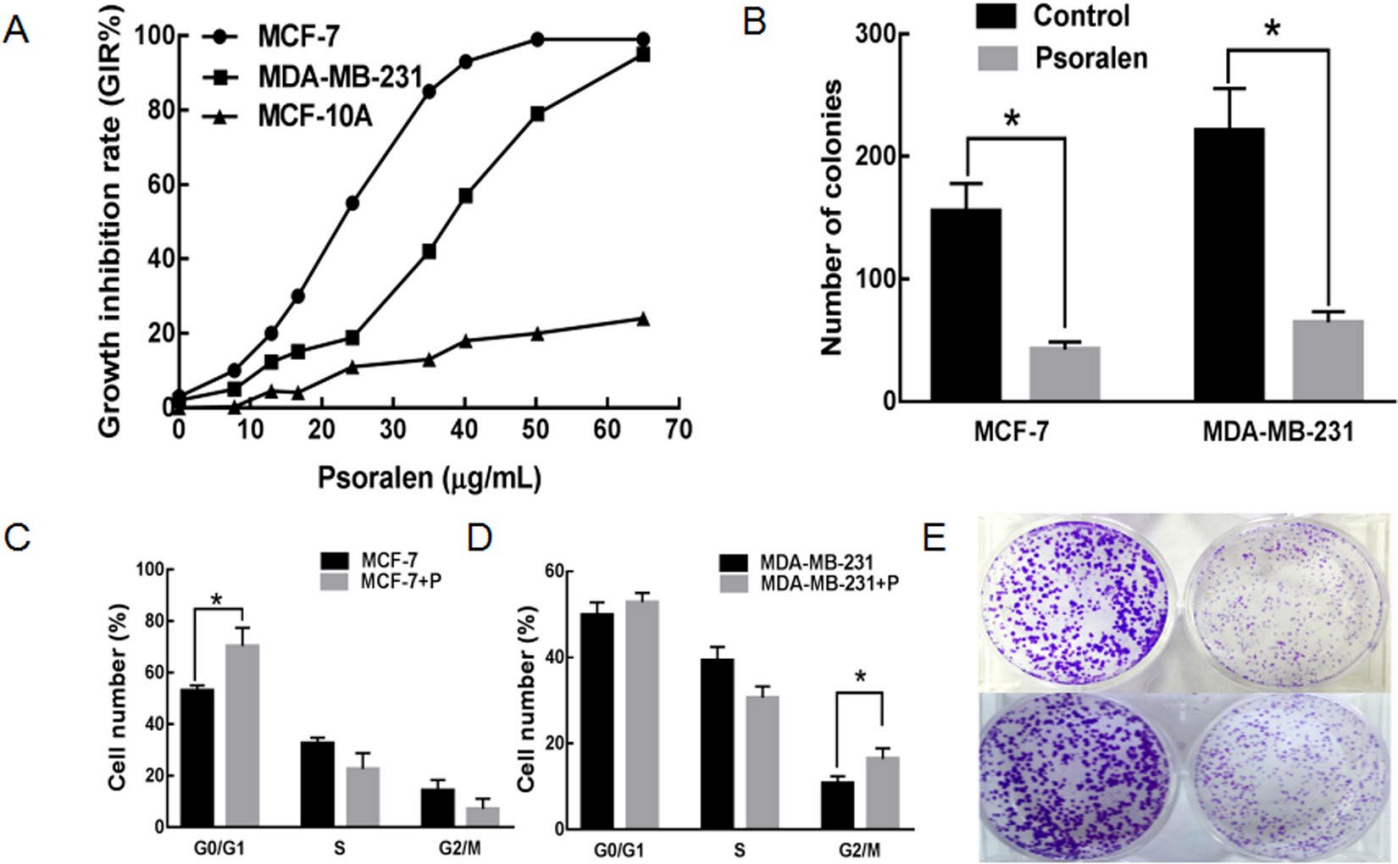
Effects of psoralen on the cytotoxicity and proliferation of breast cancer cell lines. (**A)** MTT assay was performed after MCF-7 and MDA-MB-231 cells were treated with psoralen for 48 h. (**B**) Cells were treated with psoralen for 24 h and then the colony-formation ability was investigated after culturing at 37 °C and 5% CO_2_ for 14 days. The pictures of colonies in a 6-wall dish were taken with phase contrast microscopy (**E**). The colony formation number was shown in (**B**). Flow cytometry results showed that psoralen induced G0/G1 phase cell cycle arrest in MCF-7 cells (**C**) and G2/M phase cell cycle arrest in MDA-MB-231 cells (**D**) (*p < 0.05).

The MTT assay showed that psoralen significantly reduced the proliferation of MCF-7 and MDA-MB-231 cells in a dose-dependent manner but no such significant effect on MCF-10A cells within the concentration range of 0–65 μg/mL (Fig. 1A). The IC10 of psoralen on MCF-7 cells was 8 μg/mL and 12 μg/mL on MDA-MB-231 cells. Therefore, the concentration of IC10 was considered to be a non-cytotoxic dose chosen as the working concentration in the subsequent experiments. The colony numbers in MCF-7 cells and MCF-7 + p cells were 155.41 ± 23.45 vs 42.74 ± 6.33, while MDA-MB-231 and MDA-MB-231 + p were 221.14 ± 34.25 vs 64.30 ± 8.63. Our data demonstrated that psoralen inhibited colony formation in both MCF-7 and MDA-MB-231 cells compared with the control group at 14 days (Fig. 1B,E, p < 0.05). Further, we performed cell cycle distribution analysis by flow cytometry at 48 h. As shown in Fig. 1C,D, psoralen induced cell cycle arrest in G0/G1 phase in MCF-7 cells and the percentage of G0/G1 phase was increased by (17.32 ± 4.28)% (*p < 0.05); However, psoralen induced cell cycle arrest in G2/M phase in MDA-MB-231 cells and the percentage of G2/M phase was increased by (5.71 ± 1.68)% (*p < 0.05).

### Effects of psoralen on target Wnt/β-catenin signaling genes

**Figure 2:**
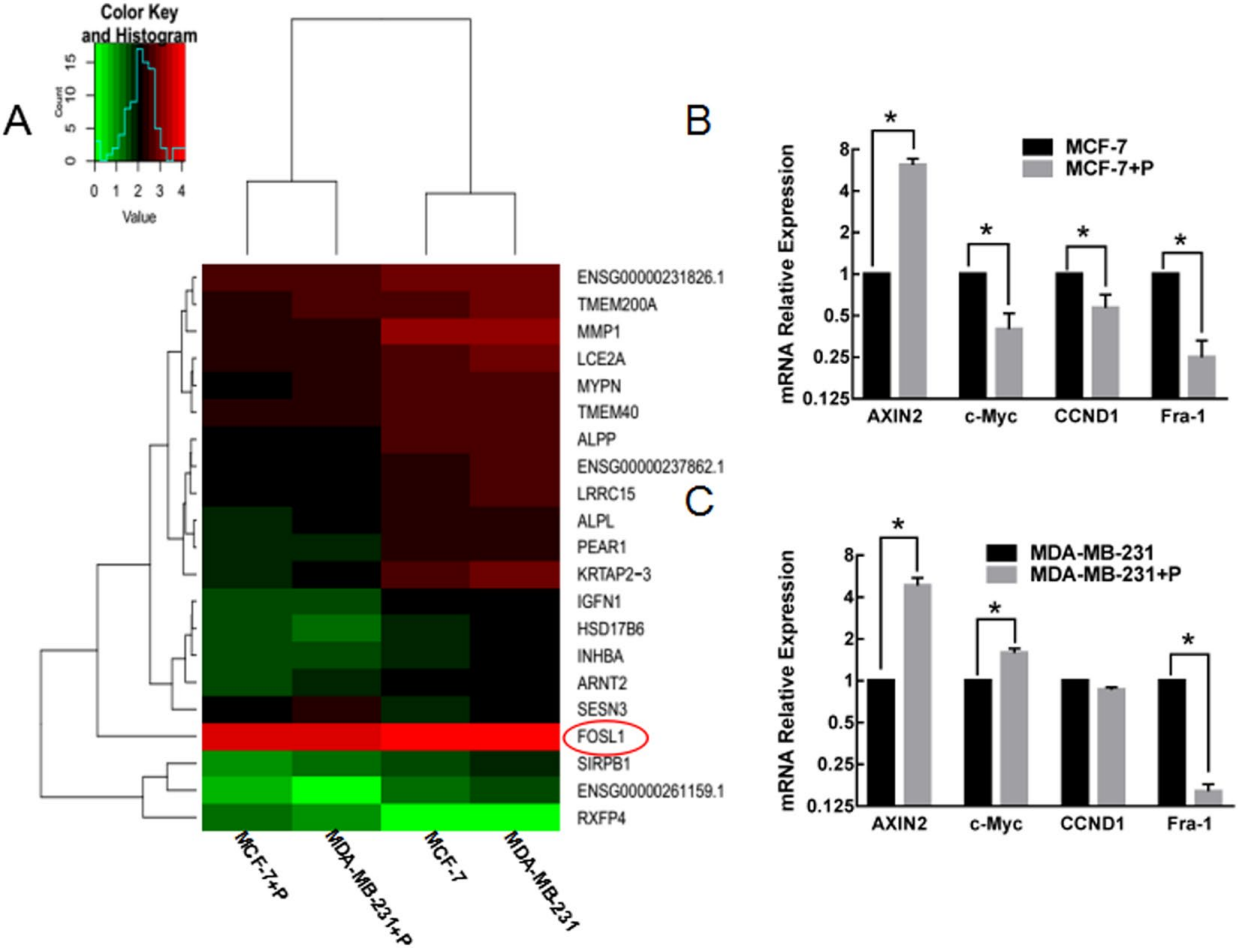
Effects of psoralen on the Wnt/β-catenin signaling target genes. (**A**) Heatmap of differentially expressed genes (DEGs) in psoralen-treated samples and control samples. (**B**,**C**) Quantitative RT-PCR of Wnt/β-catenin target genes Fra-1, cyclin D1, c-Myc and Axin2 showed the expression levels in psoralen-treated MDA-MB-231 cells and psoralen-treated MCF-7 cells as well as their control cells (*p < 0.05).

There were 2 genes were up-regulated with FI ≥ 1.50 and 19 genes were down-regulated with FI ≤ 0.50 by the RNA-Seq analysis (Fig. 2A). The GO enrichment analysis and KEGG pathway analysis indicated that Wnt/β-catenin pathway was highly enriched. Then the effects of psoralen on the expression of Wnt/β-catenin target genes (Fra-1, cyclin D1, c-Myc and Axin2) were further investigated. As shown in Fig. 2B,C, psoralen markedly reduced the mRNA expressions of Fra-1 and promoted the expression of Axin2 in both MCF-7 and MDA-MB-231 cells after treatment for 24 h. In psoralen-treated MCF-7 cells, cyclin D1 (CCND1), c-Myc and Fra-1 were all downregulated. In psoralen-treated MDA-MB-231 cells, c-Myc was upregulated, and there was no significant difference in expression for CCND1. These results further support the conclusion that psoralen could inhibit the transcriptional activity of β-catenin and subsequently lead to the suppression of Wnt target genes in breast cancer cells.

### Psoralen repressed Wnt/β-catenin pathway by reducing the expression of total β-catenin and increasing the phospho-(Y142) β-catenin

**Figure 3:**
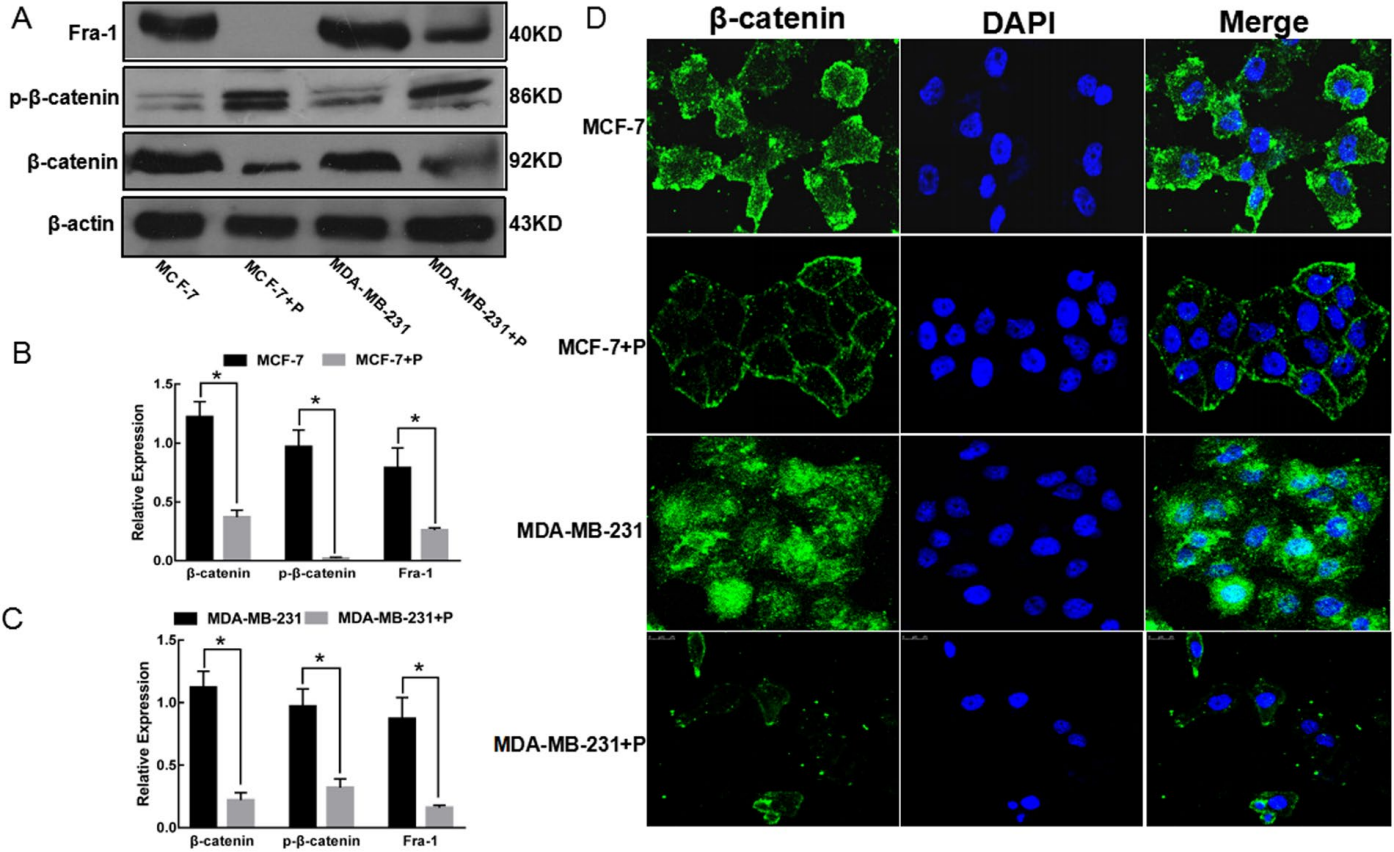
Effects of psoralen on the Wnt/β-catenin signaling. (**A**) Western blot analysis exhibited the expression of β-catenin and its downstream target gene Fra-1 in MCF-7 and MDA-MB-231 cells treated with psoralen for 72 h. (**B**,**C**) Bar graphs represent the mean normalized densitometry values of β-catenin and Fra-1 in MCF-7 and MDA-MB-231 cells, *p < 0.05 compared with the control group. Data are means ± SD of three independent experiments. (**D**) The distributions of β-catenin in the cytoplasm and nucleus were detected by immunofluorescence. The results showed strong cytoplasmic and nuclear localization of β-catenin in MCF-7 cells and MDA-MB-231 cells as well as typical membranous β-catenin expression in the cell-cell contacts after psoralen treatment.

We next investigated the psoralen regulatory effect on Wnt/β-catenin signaling by western blot and immunofluorescence. Western blot analysis revealed that the expression of the β-catenin and its downstream target Fra-1 were decreased, and the phospho-(Y142) β-catenin was up-regulated in MCF-7 and MDA-MB-231 cells after psoralen treatment (Fig. 3A–C). Immunofluorescence staining revealed a significantly reduced intracellular accumulation of β-catenin in psoralen treated cells (Fig. 3D).

### Psoralen reduced tumor growth in a xenograft model of MCF-7 cells

**Figure 4:**
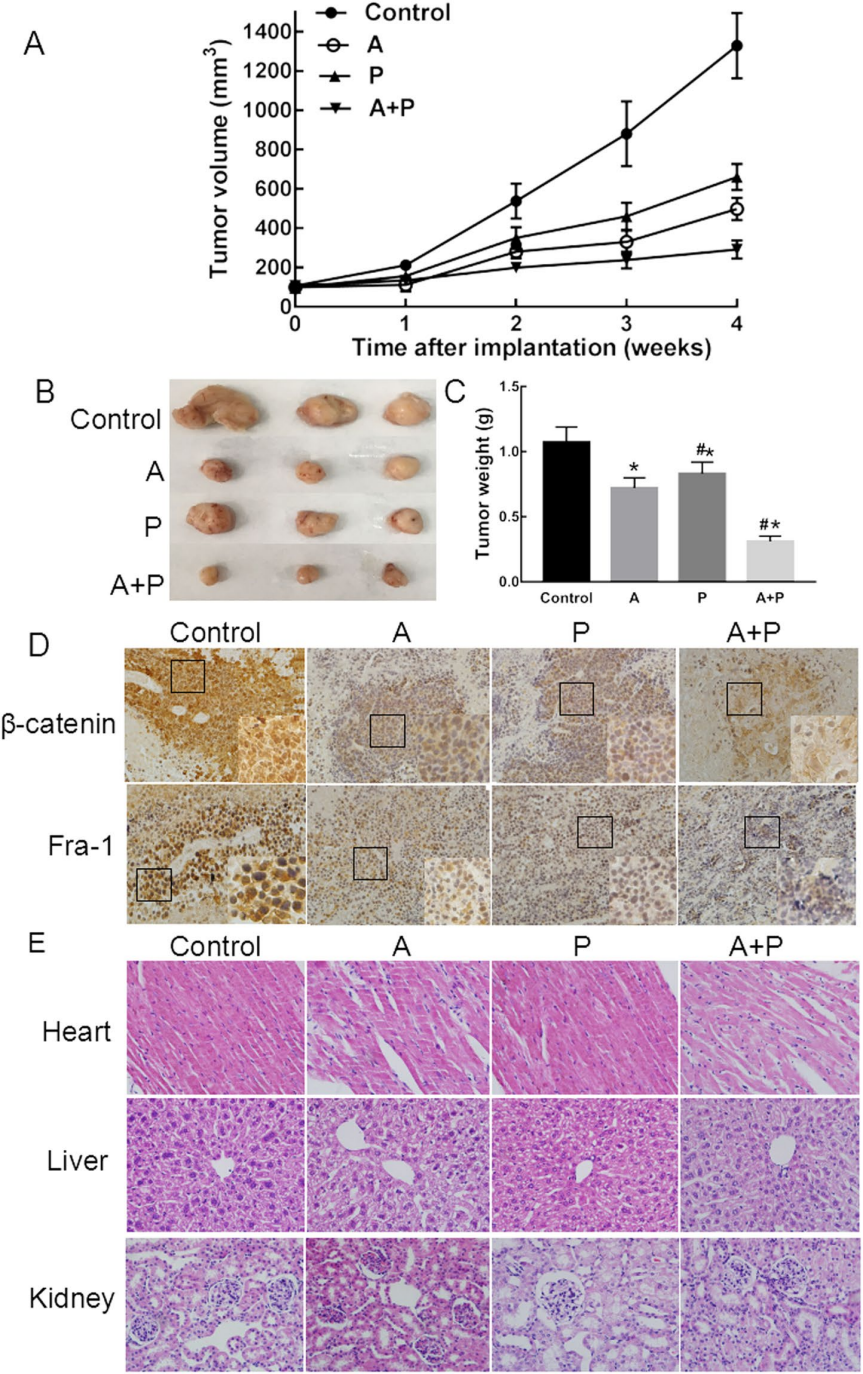
The anti-tumor effect of psoralen *in vivo*. (**A**) Tumor volume variation, *p* < 0.05. (**B**) Tumor weights of the mice groups with different treatments, *p < 0.05 vs. control group, ^#^*p* < 0.05 vs. A group. Each point represents the mean ± SD. (**C**) Representative images of tumors isolated from the xenograft model after 28 days. (**D**) Immunohistochemical analysis for the expression of β-catenin and Fra-1 (magnification, 400x) for mice of all groups. (**E**) Histopathological study of different treated groups; the heart, liver and kidneys were stained by the HE method. The scale bar is 100 μm.

The antitumor efficiency of psoralen was further validated in MCF-7 tumor-bearing mice. The tumor volume of the (A + P)-treated group was only 22% that of the control group at the end of the experiment, and the tumor volumes of mice treated with A and P were 58% and 44% those of the controls, respectively (Fig. 4A,B). The tumor weights in the control group were 1.07 ± 0.12 g. The tumors of the A, P and (A + P) groups were significantly smaller, and the weights of the tumors were 0.72 ± 0.08 g, 0.83 ± 0.09 g and 0.31 ± 0.04 g, respectively (vs. the control group, *p < 0.05); the anti-tumor effect of the A group was better than that of the P group (*#p* < 0.05) (Fig. 4C). To investigate the effects of psoralen on molecular targets *in vivo*, we further analyzed the expression levels of β-catenin and Fra-1 in the tumor samples. Immunohistochemical analysis indicated that the expression of β-catenin and Fra-1 was downregulated and their localizations were changed after psoralen treatment (Fig. 4D). The patterns of β-catenin and Fra-1 expression were mixed nuclear and cytoplasmic, but the reactivity in the cytoplasm was weaker than that of nuclear staining. As shown in Fig. 4E, β-catenin showed a loss of membranous and nuclear staining with different degrees in the A and P groups, while there was preserved membranous staining, almost without nuclear accumulation, in the (A + P) treated group. The nuclear localization of Fra-1 is important for its function as a transcription factor. Strong nuclear and weak cytoplastic immunostaining were observed in the control group. No obvious changes were observed in the A and P groups compared with the control group. However, weak cytoplasmic immunostaining and nuclear localization of Fra-1 were rarely observed in the (A + P) treated group. Psoralen at a concentration of 17.5 mg/kg did not induce significant heart, hepatic or kidney injury (Fig. 4E). Taken together, these results suggest that psoralen could inhibit MCF-7 cell proliferation *in vivo* by inhibiting the β-catenin/Fra-1 signaling pathway; thus, psoralen is a potential therapeutic candidate for breast cancer.

## Discussion

Over the past few decades, psoralen has been viewed as an attractive drug for the induction of anti-proliferation, apoptosis, cell cycle arrest and differentiation in human cancer cells, and it has acted as an effective anti-tumor agent in animal trials. Recent studies reported the anti-tumor effects of psoralen on bladder cancer, mucoepidermoid carcinoma and breast cancer. However, the mechanism of its anticancer effects and the determination of an efficacious and safe dose of psoralen have heretofore not been deeply considered, limiting the clinical use of psoralen.

Our results showed that psoralen could induce cell cycle arrest in MCF-7 cells and MDA-MB-231 cells, which may be related to its inhibitory effect on Wnt/β-catenin transcriptional activity. The expression of Wnt/β-catenin target genes, such as CCND 1 and c-Myc, was differently regulated in MCF-7 cells and MDA-MB-231 cells after psoralen treatment. Fra-1 was downregulated in both of the psoralen-treated MCF-7 and MDA-MB-231 cells, which was also consistent with our RNA-Seq results. Among the AP-1 components, Fra-1 has hitherto been generally overlooked. Fra-1 may also play an active role in mitotic progression and play a vital role in tumor initiation and progression, making it a therapeutic target^13–16^. However, there is still no ideal targeted drug for Fra-1 due to the absence of readily targeted catalytic sites. Our RNA-Seq analysis revealed that Fra-1 (FOSL1) was significantly reduced after psoralen treatment in the MCF-7 and MDA-MB-231 cells. Fra-1 was a direct target gene of Wnt/β-catenin signaling; therefore, we turned our attention to the effect of psoralen on the activity of Wnt/β-catenin signaling. It is known that β-catenin is the key transcriptional activator of canonical Wnt signaling in the nucleus. When the Wnt signal is cascaded, β-catenin will translocate from the cytoplasm to the nucleus, after which it will bind to TCF/Lef and activate target genes. In this study, we demonstrated that psoralen could significantly limit the activation of β-catenin and downregulate the expression its target gene, Fra-1.

Psoralen has been widely used as a traditional Chinese medicine due to its pharmacological activities, including its anti-tumor^17^, anti-inflammatory^18^, antipyretic and antibacterial activities. The transductional pathways activated by psoralen in target cells are still not well known today. The growing interest in the anti-tumor molecular mechanisms of psoralen has led to the identification of the main signals. However, previous studies have confirmed that psoralen could induce liver toxicity, limiting its clinical application^19^. In this study, we also provided useful information for balancing a safe and rational dose of psoralen in breast cancer treatment. The morphologic changes seen in the pathological sections of tissues were observed by 2 different pathologists, and we found that psoralen did not cause significant toxicity to the heart, liver or kidneys at the effective concentration.

Our study investigated the anti-tumor effects and mechanism of psoralen in breast cancer cells and provided new insights into the role of psoralen in clinical application. The combined application of the natural product psoralen with the traditional chemotherapeutic agent adriamycin could lower the required dose of chemotherapy and improve the anti-tumor effect.

## Materials and Methods

### Cell lines

MCF-7 and MDA-MB-231 cell lines were purchased from Nanjing KGI (Co. Ltd, Nanjing, China).

### Animals

We used 4–6 week-old female nude mice, weighting 14–18 g, purchased from the SPF Laboratory Animal Center of Beijing Vital River Laboratory (No. 11400700167348).

### Cell culture

The MDA-MB-231 cells were cultured in L-15 medium supplemented with 10% FBS in air 100%. MCF-7 cells were maintained in RPMI-1640 containing 10% FBS at 37 °C and 5% CO_2_. All cell lines were routinely tested using a mycoplasma-contamination kit (R&D).

### MTT assay to detect cell proliferation

The effects of psoralen on cell proliferation were measured by MTT assay according to our previous test method. MDA-MB-231cells and MCF-7 cells were cultured in 96-well plates with 2 × 10^4^ cells per well overnight. After treatment with varying concentrations of psoralen for 48 h, 20 μL of 3-(4,5-dimethylthiazol-2-yl)-2,5-diphenyl-tetrazolium bromide (MTT, 5 mg/ml, Sigma) was added to each well and then incubated at 37 °C for 4 h. Absorbance values were then measured at 490 nm using a microplate reader (Bio-Red).

### Plate colony formation assay

The cells were cultured in 6-well plates with 150–250 cells/well overnight. MDA-MB-231 cells and MCF-7 cells were respectively incubated with psoralen at their IC10 concentration (8 μg/mL and 12 μg/mL) for 14 days. The cells were washed twice with PBS and stained with 0.1% crystal violet (Beyotime Institute of Biotechnology) for 5 min. The number of colonies >50 cells/colony were defined as positive colonies. Triplicate wells were set up for each condition.

### Cell cycle analysis

For cell cycle analysis, cells were plated at a density of 5 × 10^4^ cells/well in 6-well plates, and allowed to grow for 24 h until 70% confluence. Cells were starved in serum-free medium for 24 h to achieve synchronization and respectively incubated with psoralen at IC10 concentration for 48 h. Then cells were harvested and fixed with 70% ethanol. Cellular DNA content from each sample was determined by cell cycle kit (KeyGen Biotech Co, Ltd). Data was collected by BD FACS Calibur (BD Bioscience USA) and analyzed using the ModFit LT 3.2 software.

### RNA-Seq and bioinformatic analysis

Total RNA were respectively extracted from MCF-7 cells and MDA-MB-231 cells using TRIzol reagent after psoralen treatment for 24 h. RNA library construction was performed using the NEBNext® Poly (A) mRNA Magnetic Isolation Module from Illumina (San Diego, CA, USA). The cDNA fragments were sequenced by the Illumina HiSeq3000. The differential gene-expression values for each sample were calculated by DEseq based on the RPKM (reads per kilo bases per million reads method). A corrected P-value of 0.005 and a log2 (fold-change) of 1.5 were set as the thresholds for significantly differential expression. The gene ontology (GO) enrichment analysis was implemented by the GO seq R package and the Kyoto Encyclopedia of Genes and Genomes (KEGG) pathways with a corrected *p* ≤ 0.05 were considered to be significantly enriched by DEGs.

### Reverse transcription and real-time quantitative PCR

The expression of Axin2, a negative regulator of the Wnt/β-catenin/TCF signaling pathway and its downstream target genes c-Myc, CCND1 and Fra-1 were assessed by RT-PCR after psoralen treatment. The primer sequences were designed and supplied from Sangon Biotech Co., Ltd. (Shanghai, China) as follows: Axin2 (forward:5′-AGTCAGCAGAGGGACAGGAA-3′, reverse: 5′-GTGGACACCTGCCAGTTTCT-3′), c-Myc (forward:5′-GGACTATCCTGCTGCCAAGA-3′, reverse: 5′-CGCCTCTTGACATTCTCCTC-3′), CCND1 (forward:5′-CCTGTCCTACTACCGCCTCA-3′, reverse:5′-TCCTCCTCTTCCTCCTCCTC-3′), Fra-1 (forward:5′-TGACCACACCCTCCCTAACT-3′, reverse: 5′-CTGCTGCTACTCTTGCGATG-3′) and β-actin (forward:5′-CCTGGCACCCAGCACAAT-3′, reverse: 5′-GGGCCGGACTCGTCATAC-3′). The RT-PCR reaction was performed with the SYBR green detection system (Thermo Scientific, Waltham, MA, USA) and the data were analyzed by 2^−ΔΔCt^.

### Western blot analysis and Immunofluorescence

To further explore the effects of psoralen on the transcriptional activation of Wnt/β-catenin signaling pathway, western blot analysis was conducted according to the previous protocol^20^. Using β-actin as an internal reference, the membrane was subsequently incubated at 4 °C overnight with primary antibodies against β-catenin, phospho-β-catenin and Fra-1 diluted at 1:3000, β-actin diluted at 1:5000 (Proteintech Group, CHI, USA), and enhanced chemiluminescence (ECL) plus kit (Millipore, America) was applied for visualization. The β-catenin expression and intracellular localization were analyzed using immunofluorescence. MDA-MB-231 cells and MCF-7 cells were fixed and stained with β-catenin antibodies (1:200 dilution). Images were captured by fluorescent microscopy.

### Tumor growth inhibitory effects of psoralen *in vivo*

Female BALB/c nude mice were kept under a 12 h light/dark cycle at the Animal Care Facility. The animals were given daily fresh diet with free access to water and acclimatized for at least 5 days prior to the experiments. All methods were approved by the Institutional Animal Care and Use Committee of Binzhou Medical University Hospital (No. SYXK-20130019). All experiments were conducted in accordance with the guidelines of the Ministry of Health of PR China and the Animal Care Committee of Binzhou Medical University. Subcutaneous tumor models were generated by injection of 1 × 10^6^ MCF-7 cells and 2 × 10^6^ MDA-MB-231 cells in medium with 50% Matrigel into the right axilla of nude mice^21^. When the tumors reached 100 mm^3^, the mice were randomly divided into 4 groups (6 mice per group): normal saline group (Control); once weekly with intraperitoneal adriamycin (4 mg/kg) group^21^ (A); twice weekly with oral psoralen (17.5 mg/kg) group^22^ (P); adriamycin combined psoralen group (A + P). At the end of experiment (28 days), mice were sacrificed and tumors were excised, weighted and photographed. The tumor volume was calculated using the formula (TV) = L × W^2^/2, where length (L) was the longest diameter and width (W) was the shortest diameter perpendicular to length.

In addition, tumor samples were removed from every group mice. The expression of β-catenin and Fra-1 was assessed by SP immunohistochemical method using rabbit-anti-human monoclonal antibody and an Histostain™- SP Kit (SPN 9001 ZSGB-BIO). The heart, liver and kidney were also collected, fixed in 10% formalin and embedded in paraffin. The paraffin-embedded tissues were sectioned at 5 μm thickness and stained with hematoxylin and eosin (H&E) for histopathological analysis.

### Statistical analyses

All data were expressed as means ± SD. They were analyzed by one-way analysis of variance (ANOVA). Differences among the treatment groups were assessed by LSD-t test, using Graph Pad Prism version 7.0 (Graph Pad Software Inc, CA, and USA). Values of *p* < 0.05 were considered to be significant.
